# Prophylactic treatment of chronic renal disease in patients undergoing peritoneal dialysis and colonized by *Staphylococcus aureus*: a systematic review and meta-analysis

**DOI:** 10.1186/s12882-016-0329-0

**Published:** 2016-08-15

**Authors:** Cibele Grothe, Mônica Taminato, Angélica Belasco, Ricardo Sesso, Dulce Barbosa

**Affiliations:** 1 Paulista School of Nursing, Federal University of São Paulo - EPE/UNIFESP, R. Napoleão de Barros 754, São Paulo, 04024-002 Brazil; 2 Division of Nephrology, Paulista School of Medicine, Federal University of São Paulo - EPM/UNIFESP), R. Botucatu 740, São Paulo, 04023-900 Brazil

**Keywords:** *Staphylococcus aureus*, Colonization, Infection, Peritoneal dialysis, Treatment

## Abstract

**Background:**

This study was performed to evaluate the clinical effectiveness of alternative strategies for the prevention and treatment of patients with chronic kidney disease undergoing peritoneal dialysis and colonized by *Staphylococcus aureus*.

**Methods:**

A systematic review and meta-analysis were performed. The literature search involved the following databases: the Cochrane Controlled Trials Register, Embase, LILACS, CINAHL, SciELO, and PubMed/Medline. The descriptors were “*Staphylococcus aureus*,” “MRSA,” “MSSA,” “treatment,” “decolonization,” “nasal carrier,” “colonization,” “chronic kidney disease,” “dialysis,” and “peritoneal dialysis.” Randomized controlled trials that exhibited agreement among reviewers as shown by a kappa value of >0.80 were included in the study; methodological quality was evaluated using the STROBE statement. Patients who received various antibiotic treatments (antibiotic group) or topical mupirocin (mupirocin group) were compared with those who received either no treatment or placebo (control group). Patients in the antibiotic group were also compared with those in the mupirocin group.

**Results:**

In total, nine studies involving 839 patients were included in the analysis, 187 (22.3 %) of whom were nasal carriers of *S. aureus*. The probability of *S. aureus* infection at the catheter site for peritoneal dialysis was 74 % lower in the mupirocin than control group (odds ratio [OR], 0.26; 95 % confidence interval [CI], 0.14–0.46; *p* < 0.001), 56 % lower in the antibiotic than control group (OR, 0.44; 95 % CI, 0.19–0.99; *p* = 0.048), and 52 % lower in the mupirocin than antibiotic group (OR, 0.48; 95 % CI, 0.21–1.10; *p* = 0.084). The difference in the probability of *S. aureus* peritonitis in patients undergoing peritoneal dialysis was not statistically significant among the three groups.

**Conclusions:**

Mupirocin and topical antibiotics were effective for reduction of *S. aureus* catheter site infection in patients undergoing peritoneal dialysis when compared with no treatment or placebo. However, evidence was insufficient to identify the optimal agent, route, or duration of antibiotics to treat peritonitis.

**Electronic supplementary material:**

The online version of this article (doi:10.1186/s12882-016-0329-0) contains supplementary material, which is available to authorized users.

## Background

Peritoneal dialysis (PD) is an effective form of renal replacement therapy in patients with end-stage chronic renal failure. Despite recent improvements in connection technology, the development of skin infection at the catheter exit site and peritonitis remains a major cause of morbidity, hospitalization, catheter removal, termination of PD with permanent transfer to hemodialysis, and even death [1].

In 2005, the International Society for Peritoneal Dialysis determined that the peritonitis rate should be fewer than 0.67 episodes per patient per year [2], and in 2011 recommended that a rate of fewer than 0.36 episodes per year should be achieved by most programs [3]. However, rates of 0.63 to 1.66 episodes of peritonitis per patient per year and a catheter exit site infection rate of 0.72 episodes per year at risk have been reported [4, 5].

Worldwide, gram-positive cocci such as *Staphylococcus epidermidis*, coagulase-negative *Staphylococcus,* and *S. aureus* are the most frequent etiological agents in peritonitis associated with PD [2]. According Barreti et al. [6] the main causative agent of peritonitis in the world is coagulase-negative *Staphylococcus* however *S. aureus* is associated with the most severe episodes and increased risks of hospitalization, death, and catheter removal [7]. A rate of *S. aureus* peritonitis of 0.12 episodes per patient per year has been reported [4].

Approximately 20 % of healthy people are chronic carriers of *S. aureus*, 30 % are intermittent carriers, and 50 % are not susceptible to carriage for unknown reasons [8]. An estimated 2 million individuals in the Netherlands are chronic carriers of *S. aureus* based on the prevalence rate in that country. In the United States, an estimated 53 million people are chronic carriers of *S. aureus* [8]. Aktaş et al. [9] demonstrated a clear association between *S. aureus* carriage and *S. aureus* infection in patients undergoing PD. Twenty-three genotypes were established for the 28 isolates, demonstrating high clonal heterogenecity. Six clinical isolates from four patients undergoing hemodialysis and four clinical isolates from two patients undergoing PD were molecularly evaluated to compare isolates obtained from infection with the carriage isolates of the same patients. All but one of these clinical isolates were "indistinguishable/closely related" to the isolates obtained from the same patients as the carriage isolates.

Various antimicrobial agents have been used for prevention of infection associated with *S. aureus* nasal colonization and peritoneal catheter exit site infection in patients with renal insufficiency. Mupirocin is an antibiotic that has been used locally in the nasal cavity and skin to eradicate *S. aureus* colonization with good results. Research conducted at the Federal University of São Paulo demonstrated that the application of topical mupirocin at the catheter insertion site significantly diminished the risk of *S. aureus* colonization and infection [10]. However, the emergence of multiresistant strains to this drug should be considered, as shown in a study conducted in Spain. That study revealed strains with high levels of resistance to mupirocin that were associated with a methicillin-resistant *S. aureus* (MRSA) pandemic [11].

In addition, systemic antibacterial agents such as ciprofloxacin, novobiocin, trimethoprim/sulfamethoxazole, and rifampicin have also been used to eradicate colonization by *S. aureus* in patients with chronic renal failure. Among these antibiotics, rifampicin has been the most studied drug for this purpose [12].

Collection of surveillance cultures can be used to control the spread of multidrug-resistant pathogens. Early detection of patients colonized with multidrug-resistant microorganisms can allow for effective establishment of measures to control cross transmission. Eradication of the *S. aureus* carrier state includes prevention of infection and transmission. Several eradication strategies have been evaluated, but studies differ significantly in their design.

To fill some gaps in this regard, we considered it important to conduct a systematic review to evaluate the clinical effectiveness of alternative strategies for the prevention and eradication of *S. aureus* carriage in patients undergoing PD.

## Methods

This systematic review and meta-analysis followed the steps proposed by the Cochrane Collaboration [13] and was performed in accordance with the PRISMA guidelines statement for systematic review reporting [14]. The inclusion criterion was *S. aureus* colonization in patients with chronic kidney disease undergoing PD as the primary outcome. The exclusion criteria were nonrandomized studies, letters, editorials, and case reports; studies involving patients <18 years of age; evaluation of *S. aureus* infection treatment outcomes without evaluation of the effect of nasal colonization; and no specification of the therapy administered to the treatment group. The two interventions compared in this meta-analysis were prophylactic treatment/decolonization to control cross transmission and *S. aureus* infection (peritonitis and skin infection at the catheter insertion site) between treated and untreated patients undergoing PD.

The following characteristics of each study were extracted: study design; total numbers of patients receiving various antibiotic treatments (antibiotic group), mupirocin (mupirocin group), and placebo or control (control group) with corresponding rates of eradication; colonization by *S. aureus*; and peritonitis.

### Study identification strategy

Relevant studies published from January 1989 to January 2014 were identified through a search of the following electronic databases: the Cochrane Library (including the Cochrane Controlled Trials Register), Embase, LILACS, SciELO, CINAHL, and Medline/PubMed. The principal descriptors used in the search were “*Staphylococcus aureus*,” “MRSA,” “MSSA,” “treatment,” “decolonization,” “nasal carrier,” “colonization,” ”chronic kidney disease,” “dialysis,” and “peritoneal dialysis.”

### Study selection

The studies were read by two independent reviewers (C.G. and M.T.) to ascertain whether they fulfilled the inclusion criteria. The reviewers were not blinded. Each reviewer evaluated the titles and abstracts of all identified studies and obtained complete photocopies of all relevant articles. In cases of doubt or disagreement, a third reviewer (D.A.B.) was solicited to issue an opinion regarding whether the study should or should not be included.

### Evaluation of methodological quality

Methodological quality was defined as confidence that the study design and reporting were free of bias. Two independent reviewers used the recommendations of the Cochrane framework and the STROBE statement (*ST*rengthening the *R*eporting of *OB*servational studies in *E*pidemiology). Based on the STROBE recommendations [13], studies included in the meta-analysis were divided into three categories: (A) >80 % compliance with the STROBE criteria, (B) 50 % to 80 % compliance with the STROBE criteria, and (C) <50 % compliance with the STROBE criteria (Table 1).

**Table 1:** Quality of clinical trials included in the present meta-analysis

Concealment of allocation	Blinded investigator	Blinded participant	Blinded assessor	Blind data analysis	Intention-to-treat	Lost to follow-up
Adequate: 5	Yes: 4	Yes: 4	Yes: 3	Yes: 1	Declared: 3	Yes: 6
Inadequate: 1	No: 2	No: 1	No: 1	No: 1	Not declared: 6	No: 0
Obscure: 3	Obscure: 3	Obscure: 4	Obscure: 5	Obscure: 7		Obscure: 3

### Data extraction and statistical analysis

The studies were initially stratified according to their design. Based on these results, they were subsequently stratified following the Cochrane methodology. Comprehensive Meta-Analysis software was used for statistical analysis. For dichotomous variables, the odds ratio (OR) with 95 % confidence interval (CI) was calculated using random-effects and fixed-effects models. The Mantel–Haenszel chi-squared test and the I^2^ test were used to calculate heterogeneity [15].

## Results

**Fig. 1:**
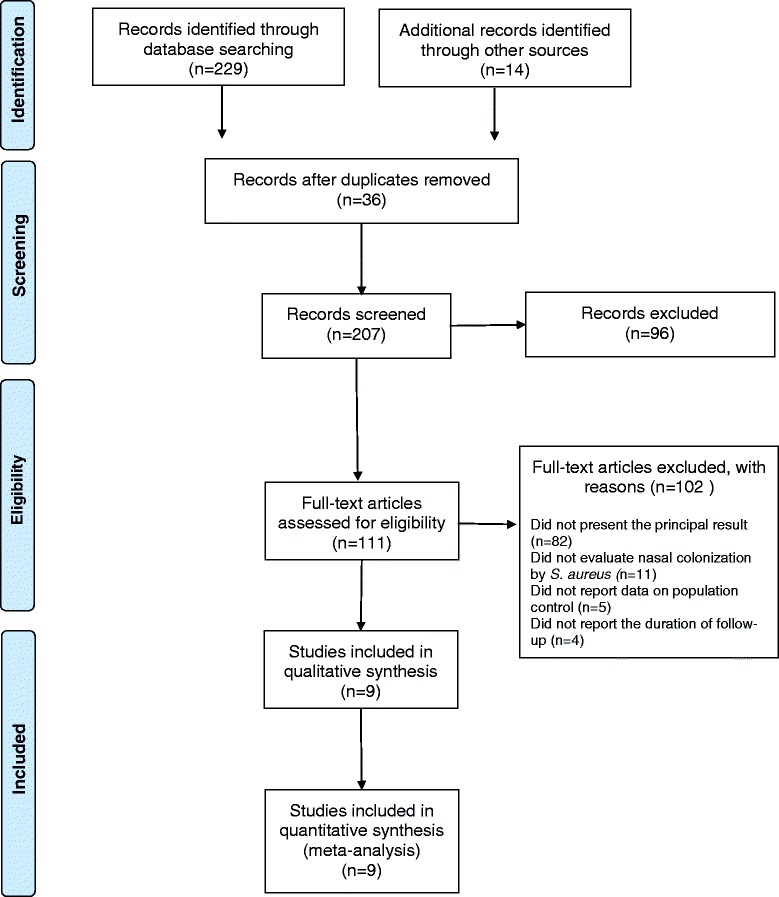
PRISMA flow diagram of systematic review inclusion and exclusion process

The PRISMA flow chart in Fig. 1 summarizes the search process. Initially, 143 articles were identified in the PubMed/Medline database, 54 in SciELO, 32 in Cochrane, 4 in LILACS, and 10 in Embase. Of the 243 total studies identified, 234 were excluded (36 were articles published and duplicated in different databases, 96 met the exclusion criteria, 82 did not present the principal result, 11 did not evaluate nasal colonization by *S. aureus*, 5 did not report data on population control, and 4 did not report the duration of follow-up).

Thus, nine studies were evaluated: a prospective double-blind randomized controlled trial [16] and eight randomized clinical trials [17–24]. All studies were evaluated and classified as having a low risk of bias and adequate methodological quality by the Cochrane referential [13]. Randomization of the studies included in this review was performed by a computer, and concealment of the allocation was adequate.

These studies involved a total of 839 individuals (420 patients in the intervention group and 419 in the control group). Of these 839 individuals, 187 (22.3 %) were nasal carriers of *S. aureus* (see Additional file 1).

Based on the STROBE recommendations [13], eight studies were placed in category A and one study was placed in category B. This meta-analysis did not include studies in category C (<50 % compliance with the criteria established by STROBE) (Table 1).

Different interventions were evaluated, including nasal agents (calcium mupirocin ointment 2 % [16, 22], nasal neomycin ointment 0.1 % [22], and sodium fusidate [20]), topically applied antimicrobial agents in the catheter exit site (calcium mupirocin ointment 2 % [17, 21, 23, 24], gentamicin sulfate 0.1 % [21], cefazolin + gentamicin [19], and sodium fusidate [20]), and orally administered antibiotics (rifampicin 600 mg [18, 23] and ofloxacin [20]) (Table 2).

**Table 2:** Screening and treatment of chronic renal patients colonized with S. areus on peritoneal dialysis

Author	Year	Design	Pacient (n.)		NCSA (n./%)		Treatment		Follow up		Erradication	ESI		Peritonitis	
			Treated group	Control group	Treated group	Control group	Treated group	Control group	Treated group	Control group		Treated group	Control group	Treated group	Control group
Mupirocin Study Group [16]	1996	DCR	134	133	18	24	Nasal mupirocin 2x/day for 5 days every 4 weeks	Placebo	18 months	18 months	90 %	14	44	18	24
Wong [17]	2003	ECR	73	81	16	14	CESS mupirocin 1x/d	No treatment	5 months	5 months	NT	0	10	1	1
Zimmerman [18]	1991	ECR	32	32	9	8	Oral rifampicin 300 mg 2x/d for 5d every 3 months	No treatment	10 months	12 months	NT	3	12	3	5
Lye [19]	1994	ECR	41	105	3	6	Cefazolin + CESS gentamicin	No treatment	36 months	36 months	NT	2	4	2	0
Sesso [20]	1998	ECR	9	13	5	5	Nasal and CESS sodium fusidate	No treatment	7,8 months	7,8 months	43 %	3	5	1	6
Sesso [20]	1998	ECR		13	5	5	Oral Ofloxacin (dd)	No treatment	7,8 months	7,8 months	40 %	2	5	4	6
Bernardini [21]	2005	ECR	67	66	9	9	CESS mupirocin 2x/d for 5d every 3 months	CESS gentamicin 2x/d for 5d every 3 months	8 months	8 months	97 %	0	10	1	1
Fontán [22]	1993	ECR	12	10	12	10	Nasal mupirocina for 7 d	Nasal neomicin for 7 d	9,5 months	9,5 months	100 %	1	1	0	1
Bernardini [23]	1996	ECR	41	41	18	18	CESS mupirocin daily	Oral Rifampicin 600 mg 1x/d for 5d every 3 months	months	12 months	NT	5	6	2	1
Cavdar [24]	2004	ECR	18	18	3	0	CESS mupirocin 3x/week	CESS mupirocin 1x/week	6 months	6 months	83 %	0	1	0	1

Note: *n* number of patients, *%* Percentage, *NCSA* nasal carrier of S. aureus, *CESS* catheter exit site skin, *DBRCT* Double blind randomized controlled trial, *RCT* randomized clinical trial, *NT* not tested, *ESI* Catheter Exit Site Infection, *3x/w* three times per week

Several therapies were compared to evaluate the infection prevention effectiveness at the exit of the PD catheter and peritonitis caused by *S. aureus*. Mupirocin was compared with no treatment or placebo in two studies [16, 17]. Mupirocin was compared with gentamicin, rifampicin, and neomycin in four studies [21–24]. The antibiotics rifampicin, cefazolin + gentamicin, ofloxacin, and sodium fusidate were compared with placebo or no treatment in three studies [18–20].

**Fig. 2:**
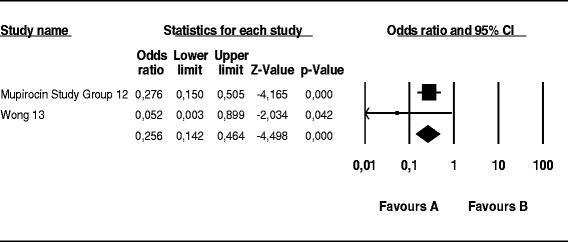
Mupirocin treatment *versus* placebo or no treatment in patients on peritoneal dialysis colonized by *S. aureus*: Number of patients with CESI

**Fig. 3:**
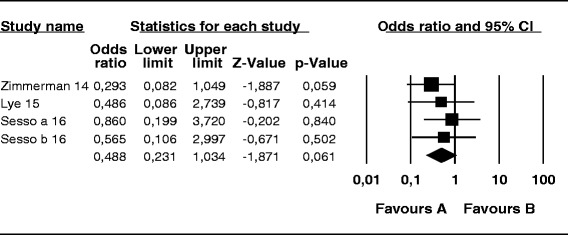
Antibiotics treatment *versus* placebo or no treatment in patients undergoing peritoneal dialysis colonized by *S. aureus*: Number of patients with CESI

**Fig. 4:**
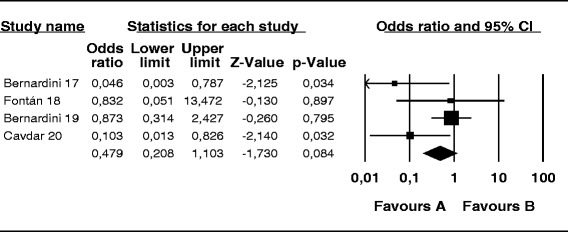
Treatment with mupirocin vs. antibiotics in peritoneal dialysis patients colonized with *S. aureus*: Number of patients CESI

The probability of skin infection at the PD catheter exit site caused by *S. aureus* was 74 % lower in the mupirocin than control group (OR, 0.26; 95 % CI, 0.14–0.46; *p* < 0.001) (Fig. 2), 56 % lower in the antibiotic than control group (OR, 0.44; 95 % CI, 0.19–0.99; *p* = 0.048) (Fig. 3), and 52 % lower in the mupirocin than antibiotic group (OR, 0.48; 95 % CI, 0.21–1.10; *p* = 0.084) (Fig. 4).

**Fig. 5:**
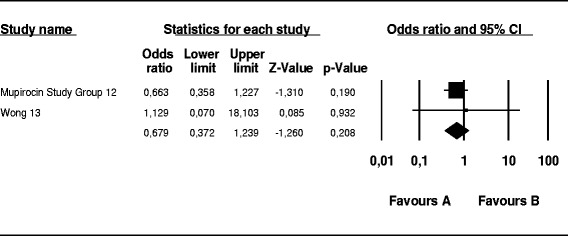
Mupirocin treatment *versus* placebo or no treatment in patients on peritoneal dialysis colonized by *S. aureus*: Number of patients with peritonitis

**Fig. 6:**
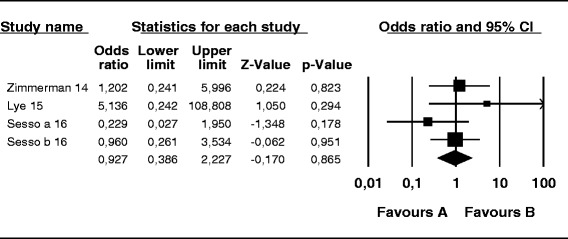
Antibiotic treatment *versus* placebo or no treatment in patients on peritoneal dialysis colonized by *S. aureus*: Number of patients with peritonitis

**Fig. 7:**
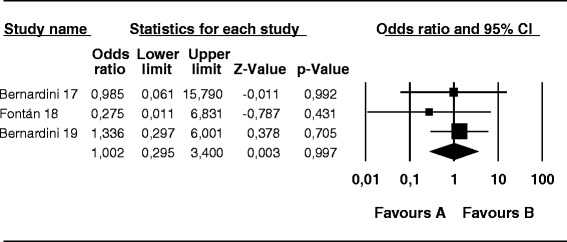
Treatment with mupirocin *versus* antibiotics in peritoneal dialysis patients colonized with *S. aureus*: Number of patients with peritonitis

The difference in the risk of peritonitis caused by *S. aureus* in patients undergoing PD was not significant among the three groups: mupirocin versus control group (OR, 0.68; 95 % CI, 0.37–1.24; *p* = 0.20) (Fig. 5), antibiotic versus control group (OR, 1.072; 95 % CI, 0.43–2.69; *p* = 0.88) (Fig. 6), and mupirocin *versus* antibiotic group (OR, 0.74; 95 % CI, 0.18–3.05; *p* = 0.67) (Fig. 7).

Table 2 provides the following details: author, year of publication, study population, total number of included patients, number of patients colonized with *S. aureus* at study entry, number of episodes of infection at the catheter exit site peritoneal dialysis; and number of episodes of peritonitis (number of episodes over total months in patient therapy).

## Discussion

The rates of nasal *S. aureus* colonization, skin infection at the exit site of the PD catheter, and peritonitis were studied in this review. The review included 839 patients with chronic kidney disease undergoing PD, 22.3 % of which were *S. aureus* nasal carriers.

Decolonization therapy may involve topical treatment by use of drugs at the catheter exit site or systemic antibacterials by oral administration of antibiotics. However, there is no evidence to indicate what type of treatment (topical or systemic) is most effective for such a procedure.

Topical treatments are widely used and evaluated, and the main antimicrobial agents tested include calcium mupirocin ointment 2 % [16, 17, 21–24], sodium fusidate ointment 2 % [20], cream gentamicin, neomycin sulfate [22], and cefazolin cream [19]. We found that topical mupirocin reduced the risk of *S. aureus* infection at the exit site of the PD catheter by 74 % compared with the control group (placebo or no treatment) (*p* < 0.001). Corroborating this result, it appears that mupirocin-based topical treatment is indicated by most guidelines of international health agencies, including the Centers for Disease Control and Prevention (CDC) [25] and World Health Organization (WHO) [26]. The CDC recommends the use of topical mupirocin alone in patients colonized only for a short period of time and for health care professionals [25]. Conversely, the WHO considers the use of mupirocin and/or chlorhexidine in patients with MRSA, but does not specify the indications, contraindications, or time of use [26]. Notably, for the National Institute of Clinical Excellence in the UK, the use of topical antimicrobials is not indicated in any case because there is no evidence that this practice may reduce health care associated infections (HAIs) either global or those caused by MRSA [27].

Another contraindication to mupirocin is bacterial resistance to this agent. In cases involving a mupirocin-resistant agent, the possibility of endogenous recolonization is high after 4 weeks of treatment [27]. However, resistance to mupirocin is still considered low. In this review, we found only one study that reported resistance to mupirocin in one isolated case.

Gentamicin and cefazolin were used together, and this combination provided effective results to reduce infection rates at the exit site of the PD catheter (0.23 vs 0.09 episode per patient per year; *p* < 0.005) and peritonitis (0.33 vs 0.10 episodes per patient per year; *p* < 0.005) [19].

Fusidic acid was evaluated separately and exhibited statistically significant results (*p* < 0.01) [20] for *S. aureus* eradication in colonized patients. However, it is widely contraindicated as monotherapy for decolonization and is indicated only as a supportive treatment or enhancer to the use of systemic rifamycin. It did not show statistically significant results in reducing MRSA infections. Instead, it increased the emergence of bacterial resistance, which reinforces the idea of not using this drug alone for eradication of *S. aureus* in colonized patients [27].

Only one study compared the effectiveness of nasal neomycin sulfate ointment three times a day for 7 days with that of nasal mupirocin ointment daily for 7 days and reported that mupirocin is more effective than neomycin sulfate for elimination of *S. aureus* nasal colonization in patients undergoing continuous ambulatory PD [22].

In this meta-analysis, the antibiotic group exhibited a 56 % lower risk of *S. aureus* infection at the PD catheter exit site compared with the control group (*p* = 0.048).

Systemic treatment alone for *S. aureus* eradication is not widely used; instead, systemic treatment is generally associated with a topical drug. This is mainly due to concerns about the emergence of strains resistant to antimicrobials [27]. The main antimicrobial agents for systemic use found in this study were rifamycin and ofloxacin [18, 20]. In the main guidelines of international agencies (the WHO, CDC, and British Society for Antimicrobial Chemotherapy [24–26]), the systemic treatment recommendation for decolonization is that the therapeutic drug is chosen based on consultation with medical specialists in infectious diseases and epidemiologists at hospitals.

We found that patients treated with rifampicin showed a significant delay in the time to first infection associated with the PD catheter (*p* < 0.015) and significantly fewer infections related to the PD catheter (*p* < 0.001), but rifampicin was associated with toxicity and side effects. Two studies reported treatment interruption due to toxicity in 6.6 % to 12.0 % of patients; however, no irreversible toxicity was observed, perhaps because of the short duration of treatment used in the studies, which may not have been long enough to cause significant clinical interactions [18, 23].

Another systemic medication used was oral ofloxacin, which was found in only one study and compared with the use of topical sodium fusidate ointment. Oral ofloxacin was less effective in the eradication of nasal colonization and reduction of infection at the PD catheter exit site, and both treatments showed no effect in reducing episodes of peritonitis [20].

This meta-analysis has shown that the use of topical antibiotics reduces the incidence of PD catheter exit site infection, but not the incidence of peritonitis. This may simply be a power problem (exit site infections being much more common than peritonitis, plus the relatively small numbers of patients involved), but raises the question about the relationships among carriage, infection, and peritonitis. It is likely that infection is introduced mainly at exchange via contamination of the catheter tip rather than tracking along the tunnel. A better technique might reduce the risk. Our findings are similar to those of the Cochrane Review by Strippoli et al. [15], who also concluded that nasal mupirocin reduces exit site and tunnel infections, but not peritonitis. The European Guidelines [28] state that the “use of mupirocin or gentamicin cream at the exit site is recommended to reduce exit site infections,” but cites no evidence that this reduces peritonitis. Like the other guidelines, the authors had to extrapolate from reduction in exit site infections to reduction in peritonitis.

This review and meta-analysis had some limitations. The included studies had a short follow-up and did not allow for analysis of long-term treatment, which may have substantially different efficacy and safety than shown in the present results. Additionally, heterogeneity was present between the treatments applied, and it is not possible to conclude the best strategy for the use of these antibiotics. Moreover, local protocols and patient education interventions for the prevention of infection were not analyzed in this study.

Because patients often become recolonized with *S. aureus* after receiving an initial treatment regimen, regular examinations with mupirocin application seems to be a reasonable option. Meticulous attention to infection control practices during insertion and access of the PD catheter is also of paramount importance in the prevention of *S. aureus* infection.

Future longitudinal studies need to define a mupirocin regimen that balances a reduction in the incidence of *S. aureus* infection with concerns about the emergence of mupirocin resistance.

## Conclusion

Peritonitis is the main cause of the need to change PD to hemodialysis, which reduces patients’ quality of life and increases costs to the national health system. Unfortunately, the current evidence base for the prevention of peritonitis is not supported. Nasal, oral, and local interventions reduce infections at the exit site of the PD catheter, but not peritonitis. However, this finding may be due to testing very small numbers of patients and for very short periods or because the incidence of peritonitis was low.

This meta-analysis supports the use of local antibiotics at the catheter exit site in patients undergoing PD. However, the available data are very limited, and more studies are needed to examine the clinical importance of antibiotics at the catheter exit site in patients undergoing PD.

## Abbreviations

CI, confidence interval; MRSA, methicillin-resistant *S. aureus*; MSSA, methicillin-sensível *S. aureus*; OR, odds ratio; PD, peritoneal dialysis; PRISMA, guidelines statement for systematic review reporting; *S. aureus, Staphylococcus aureus*; STROBE, strengthening the reporting of observational studies in epidemiology

## Additional file

Additional file 1: PRISMA checklist. (DOC 63 kb)
